# Internet-based relapse prevention for anorexia nervosa: nine- month follow-up

**DOI:** 10.1186/2050-2974-1-23

**Published:** 2013-07-30

**Authors:** Manfred Maximilian Fichter, Norbert Quadflieg, Susanne Lindner

**Affiliations:** 1 Department of Psychiatry and Psychotherapy, University of Munich (LMU), Nussbaumstraße 7, 80336 München, Germany; 2 Schön Klinik Roseneck affiliated with the Medical Faculty of the University of Munich (LMU), 83209 Prien, Germany

**Keywords:** Anorexia nervosa, Relapse prevention, Internet-based prevention, Online psychotherapy, Risk of relapse, Adherence, Eating disorder, Internet, Follow-up, Maintenance

## Abstract

**Background:**

To study the longer term effects of an internet-based CBT intervention for relapse prevention (RP) in anorexia nervosa.

**Methods:**

210 women randomized to the RP intervention group (full and partial completers) or the control group were assessed for eating and general psychopathology. Multiple regression analysis identified predictors of favorable course concerning Body Mass Index (BMI). Logistic regression analysis identified predictors of adherence to the RP program.

**Results:**

Most variables assessed showed more improvement for the RP than for the control group. However, only some scales reached statistical significance (bulimic behavior and menstrual function, assessed by expert interviewers blind to treatment condition). Very good results (BMI) were seen for the subgroup of “full completers” who participated in all nine monthly RP internet-based intervention sessions. “Partial completers” and controls (the latter non-significantly) underwent more weeks of inpatient treatment during the study period than “full completers”, indicating better health and less need for additional treatment among the “full completers”. Main long-term predictors for favorable course were adherence to RP, more spontaneity, and more ineffectiveness. Main predictors of good adherence to RP were remission from lifetime mood and lifetime anxiety disorder, a shorter duration of eating disorder, and additional inpatient treatment during RP.

**Conclusions:**

Considering the high chronicity of AN, internet-based relapse prevention following intensive treatment appears to be promising.

## Background

Anorexia nervosa (AN) is a serious mental disorder with very high rates for chronicity and mortality. In order to reduce chronicity and counteract mortality in AN patients, better and more effective treatments are needed. However, we also need more effective programs for maintaining an improved level of mental health that was achieved through face-to-face therapy; effective relapse prevention over longer periods of time following intensive treatment is truly essential for AN patients. Technological developments of communication media in the past years and decades have brought about new options for clinical research and practice. The development of guidelines for psychotherapy based on empirical results from RCTs (randomly controlled trials) and the development of detailed manuals for use in psychotherapy treatment studies has been very helpful for the next stage of using electronic media and the internet to convey relevant information and interventions to patients suffering from physical or mental disorders.

Internet-based programs can also reach patients or persons at risk who can only be reached with great difficulties by more traditional approaches [1]. There is hardly any limitation in the number of persons who can be reached by an internet-based program. Such programs, however, must not replace traditional service delivery; rather, they should complement and extend the options for medical and psychotherapeutic treatment in situations where there still is a great need [2].

There are a significant number of controlled internet-based studies for primary, secondary, and tertiary prevention for physical disorders, mostly delivering interventions for asthma [3], cardiological disorders [4], management of work-related stress [5], the promotion of physical activity to improve health in general [6], and diabetes mellitus [7]. Parallel to these internet-based developments concerning physical disorders, far more than a hundred randomized controlled studies utilize internet-based forms of psychotherapy in a wider sense. Moreover, there have been systematic reviews and meta-analyses concerning internet-based interventions for depression and anxiety disorders combined [8], psychological interventions in general [9], and for depression only [10]. Other controlled studies have dealt with internet-based psychotherapy for posttraumatic stress disorder [11], anxiety disorders [12] including panic disorder and various phobias, and depression [13]. Generally, results of RCTs employing internet-based forms of psychotherapy for physical as well as mental disorders have shown promising results concerning symptom reduction, number of medical consultations, number of sick days, and improvement of coping skills. Internet-based psychotherapeutic approaches can make creative use of a variety of technological options, for example of multiple colors, cartoons or moving animations, and auditory enhancements at crucial points in the process; they can be accompanied by (therapist-guided) internet chat groups, partially or fully automated e-mail and SMS-support [14,15], and standardized telephone contacts to give feedback or encourage participants to use the program conscientiously. Bauer et al. [16], for example, describe successful therapist-guided internet chat groups for relapse prevention following inpatient treatment for mood, personality, and somatoform disorders.

For eating disorders, until recently the few existing studies on internet-based interventions have focused mainly on bulimia nervosa (BN) [15,17-23] and binge eating disorder (BED) [24-27]. Meanwhile, research on internet therapy has expanded. One group addressed users’ views and the relevance of individual parts of the program [28,29], while another group compared online versus face-to-face delivery of cognitive behavioral therapy to BN patients [30]. Both studies showed favorable results.

In treatment trials for anorexic and bulimic disorders, cognitive behavior therapy (CBT) has been the most widely used form of treatment. CBT concepts did constitute the main therapeutic approach in our nine-month internet-based relapse prevention program (RP). Two CBT treatment studies for AN inspired our research in this area; however, they both were delivered face-to-face and not via internet: In a one-year trial, Pike et al. [31] found that CBT was superior to nutritional counseling. In a larger controlled study, Carter et al. [32] reported that relapse rates were lower in AN patients receiving the CBT study intervention for maintenance of therapy outcome rather than with treatment as usual (TAU).

Effects of our nine-month internet-based intervention without follow-up data in the intention-to-treat (ITT) sample have already been published elsewhere [33]. The current paper focuses on the nine-month follow-up of an internet-based randomized controlled trial for relapse prevention in adult anorexia nervosa in a sample of program completers. For the current paper we intentionally analyzed the data of participants who received at least four of nine modules of the relapse prevention program and a limited amount of additional treatment (see below) to allow valid conclusions on the use and the effects of such programs. To our knowledge, our RCT on internet-based prevention to reduce the risk of relapse for anorexia nervosa (AN) following intensive (inpatient) treatment is the first such study to address this dangerous eating disorder. Other studies applying internet-based intervention focused on caretakers of AN patients [34,35] or on the parents of female adolescents at risk for AN [36]. Another study targeted young women with body image concerns, offering a prevention program via internet [37]. AN, of all eating disorders, carries the highest rates of chronicity and mortality in adolescent and young women. It is probably *the* psychiatric disorder with the highest mortality in that age group. A controlled treatment trial with AN patients is always a challenge, because AN patients frequently do not perceive the severity of their illness, tend to avoid treatment and frequently take a more passive, not highly motivated role during treatment. Compared to other eating disorders such as bulimia nervosa (BN) and binge eating disorder (BED), very few controlled psychotherapy trials have been conducted for AN. Of the few existing controlled treatment trials for AN, some have revealed enormously high (50 %) relapse rates [38].

The intervention period of intensive treatment with approximately 90 inpatient days was followed by a nine-month follow-up period. The aim of our study was to evaluate the longer-term efficacy of our internet-based CBT relapse intervention program (RP) for AN compared to a control group of AN patients who did not receive additional treatment from us. The main focus of the statistical analyses and data presentation in this paper lies on longer-term maintenance covering the time from the end of the relapse prevention program (T2) until follow-up nine months later (T3). This RCT was registered with the German Registry of Clinical Trials (DRKS00000081) and with Current Controlled Trials (ISRCTN20173615).

## Methods

### Sample

Participants for this prospective controlled and randomized study entered the study between April 2007 and September 2009 while treated in one of eight hospitals in Germany providing specialized inpatient services and psychotherapy for eating disorders. The study included one interventional arm (relapse prevention, RP) and one control arm for comparison (controls). Inclusion criteria were a) female gender, b) a minimum age of 16 years, c) anorexia nervosa or subthreshold anorexia nervosa without the requirement of amenorrhea according to DSM-IV criteria, d) easily accessible internet connection available at home, e) at least a 2-point BMI increase during inpatient therapy if the BMI at admission was below 14, or at least one additional BMI point in patients with a BMI above 14 upon admission, f) sufficient motivation for further relapse prevention and for taking part in the study (defined as not having a history of long inpatient stays without a clinically significant weight gain or patient-initiated irregular discharges, no history of forced feeding, good compliance with psychotherapy and routine questionnaires during the index inpatient treatment, and the assumption of sufficient compliance with the RP by the individual’s therapist). Individuals with other serious mental or physical impairments, acute or chronic organic or schizophrenic psychosis, marked suicidal ideation and/or behavior, and premature, irregular discharge from inpatient treatment were excluded from the study.

The study protocol was approved by the ethics committee of the Bavarian Medical Association and the ethics committees of other relevant German states. All participants provided informed written consent before they engaged in any research activity.

**Figure 1:**
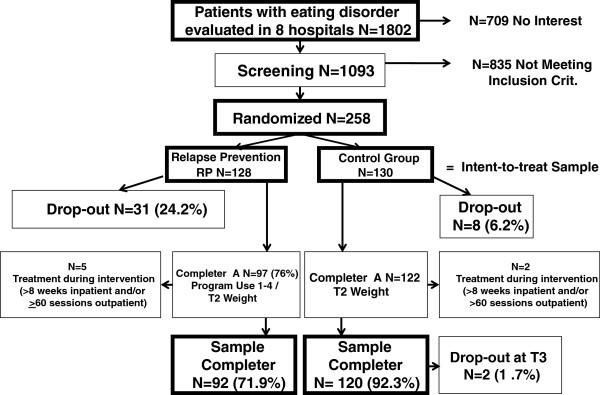
Consort diagram of sample flow.

Figure 1 presents a CONSORT diagram of the patient flow in the study. During the study period a total of 1,802 female patients with anorexia nervosa or subthreshold AN (EDNOS type 1) aged 16 years or above were treated in the participating hospitals. Of these patients, the therapists reported 1,093 to the clinical research study center at Roseneck hospital in Prien; the other patients were either not informed about the study by their therapists or did not consent to being contacted by the study team. Before randomization, a clinical psychologist examined each patient with regard to diagnosis and inclusion or exclusion criteria using questionnaires and telephone or face-to-face interviews during the last weeks of inpatient treatment. Two hundred and fifty-eight individuals expressed interest in the study and met inclusion criteria. At the end of inpatient treatment, 128 participants were randomized into the RP group and 130 participants into the control condition. Randomization was performed at the independent ‘Koordinierungszentrum für klinische Studien’ (KKS Center), Marburg, Germany.

The control group ^a)^ received no treatment whatsoever from our team throughout the study following discharge from inpatient treatment; these former patients merely filled out the questionnaires at the relevant time points. For obvious ethical reasons the research team did not interfere in any way with decisions on additional external treatment but left this entirely to the patients’ and their physicians’ or therapists’ discretion. Concomitant in- and outpatient treatments during the intervention and the follow-up periods were carefully documented for both groups (intervention group and controls). During the follow-up period, none of the patients received any intervention or other kind of therapeutic support from our side.

Of the 128 participants randomized into the RP condition, 31 participants (24.2%) *dropped out* from the RP after no or only minimal use of the program. Five other participants in this group reported a high amount of additional treatment during the course of the RP (defined as more than eight weeks of inpatient treatment or at least 60 outpatient sessions of psychotherapy). In the control group, eight participants did not provide their body weight at the end of the nine-month “intervention” period (T2), and two others met the criterion of a high amount of additional treatment during that time. A (perceived) lack of time and the avoidance of being confronted with one’s own eating attitudes and behaviors constituted the main reasons for not participating in the T2 assessment. Excluding these individuals from statistical analyses resulted in a *completer sample* of 92 participants in the RP group and 120 participants in the control group. At the nine-month follow-up (T3), two participants of the control group did not provide data. Therefore, nine-month follow-up data will be reported on 118 controls and 92 RP participants.

Of the 92 RP completers, 48 patients (52.2%) completed all nine CBT internet modules according to the protocol. Twenty-nine patients (31.5%) were partial users and worked through seven to eight sessions of the RP. Fifteen patients (18.5%) used four to six sessions.

Data presented in this paper are based on the completer sample (see Figure 1). The reasons for this are: 1. Intent-to-treat analyses concerning the intervention effects have been published already [33]. 2. The completer analysis generates valuable information about those who actually used the internet program. This allows us to view the data from a different perspective. 3. AN patients from other treatment studies tended to drop out at high rates, so information on those who (almost) completed our program will be quite useful in order to reduce sample attrition in the future. 4. In addition, results will show that a subgroup of highly motivated AN participants shows a much better outcome than completers of the control group.

### Design

For each participant, the study lasted a total of 18 months after inpatient treatment with three observation points: T1 (baseline) at the beginning of the relapse prevention program, which started right at the end of inpatient treatment, T2 (end of intervention after x weeks), and T3 (follow-up) after additional nine months. This paper mainly focuses on the time period from the end of intervention (T2) until the follow-up (T3).

### Measures

Body weight was measured by a person blind to the randomization condition (by a nurse from the treating hospital and by a general practitioner near the patients’ home at the end of the RP program and at follow-up). The body-mass index (BMI) was created by the formula: body mass in kilogram divided by the square of the person’s height in meters.

The Structured Inventory for Anorexic and Bulimic Syndromes Expert Rating (SIAB-EX) [39-43] was conducted to assess changes over time. After finishing the SIAB-EX, the interviewer rated the general severity of the anorexic eating disorder on the Psychiatric Status Rating Scale (PSR) [44]; furthermore, the Morgan Russell Outcome Assessment Schedule (MROAS) [45] focused on additional aspects of AN outcome. Interviewers at T2 (end of intervention) and T3 (follow-up) were blind to the treatment condition of the participants. These interviews were conducted by phone. Psychiatric comorbidity was assessed by the Structured Clinical Interview for DSM-IV Axis I Disorders SCID-I [46,47].

Several self-rating scales completed the questionnaire set: The Eating Disorder Inventory-2 (EDI) [48-51] targets eating specific psychopathology; the Barratt Impulsiveness Scale BIS-11 [52] and the Brief Symptom Inventory (BSI) [53,54] both measure aspects of general psychopathology. Participants also reported on newly occurring pregnancies. Baseline (T1) assessments were conducted right at the end of the inpatient treatment preceding the intervention.

### Internet-based intervention

The nine-month web-based CBT relapse prevention program for anorexia nervosa (VIA) after inpatient treatment was developed by our research team and is described in detail elsewhere [33,55]. In short, the program was created according to approved manuals, self-help manuals, and aftercare manuals based on Cognitive Behavioral Therapy (CBT) for anorexia nervosa and related disorders [31,56-62] and adapted to presentation via internet. Participants of the RP group received nine monthly online CBT-based sessions (Chapter 1: Goals and motivation; Chapter 2: Transfer of relevant therapy components conveyed during hospital treatment to everyday life, maintenance of a regular and balanced eating behavior, handling binges and compensatory behaviors; Chapter 3: Body acceptance, Chapter 4: Self-esteem; Chapter 5: Coping with emotions and coping with a variety of feelings and with emotional needs; Chapter 6: Social competence and relationships; Chapter 7: Problem solving; Chapter 8: Depression; Chapter 9: Termination and farewell. An electronic message board for peer support, monthly chat sessions, and automatic short messages complemented the program. Participants could contact the therapist via e-mail. Whenever necessary, the therapist took previously anticipated and then standardized steps to motivate the participants to use the program.

### Data analysis

A previous article [33] presented details on the rationale concerning power and sample size. In the current study, means with standard deviations and frequencies for categorical variables are reported, including t-tests for group comparisons, analyses of variance (ANOVA) and chi^2^-tests. Fisher’s exact test allowed to compare proportions between groups when any cell count in a 2 x 2 table was less than five. The longitudinal data were suitable for a factorial design with ANOVAs on time point (repeated measures factor) and on intervention condition (RP versus controls, between-subjects factor). Another ANOVA compared groups regarding treatment, and critical distances (Scheffé; 5%) were computed for post-hoc pairwise comparisons. Missing data differed slightly between measures, and for each analysis the number of cases is reported.

Data on body weight and menstrual function led to the exclusion of participants who were pregnant at the time of assessment and who otherwise would have been included in the analyses.

Two-tailed testing was applied throughout. The primary outcome variable according to the study protocol was defined as the mean difference in BMI from randomization to the end of the online intervention [33]. We subsequently extended this definition to the primary outcome at follow-up; the dependent variable thus was the mean difference in BMI from randomization to follow-up over a total time period of 18 months. Due to the fact that this follow-up study on internet-treated AN is the first in its field, no hypotheses concerning course and outcome except for the primary outcome were derived. All additional analyses were exploratory in nature and did not account for multiple testing. P-values below .10 are given in the text to allow an appraisal of relevance.

In (only) four cases at follow-up T3, the general practitioner had not been able to weigh the participants beforehand, and therefore self-reported body weight from the interview was substituted. At the end of the intervention T2, this substitution had been utilized in 3 RP and 16 control participants.

Linear regression analysis on primary outcome (weight change from T1 (baseline) to T3 (follow-up)) identified six predictors in the ITT RP sample published previously [33]. The same predictors were entered in a linear regression analysis on primary outcome defined for T3 (follow-up; BMI at T3 – BMI at T1) including 88 of 92 RP participants in order to gain insight into the longer-term power of predictors of successful relapse prevention.

A range of eating disorder relevant variables known from the literature [63] were employed as predictors at baseline in logistic regression analyses on adherence to the RP. Possible predictors included age, duration of inpatient stay before RP, age at onset of eating disorder, duration of eating disorder, body weight at admission to inpatient treatment and at the beginning of the RP, type of AN (restricting or binge eating/purging type), psychiatric comorbidity, adherence to the RP (using all nine sessions versus others), use of psychiatric drugs during RP, additional inpatient treatment during and after RP, symptom severity of eating disorder, and impulsiveness. Forward selection allowed to reduce the large number of potential predictors.

Full completers who went through all nine sessions of the RP (N = 48) were classified as showing good adherence, and partial completers (N = 44) were classified as showing poor adherence to the RP. Newly defined variables of remitted mental illness were added to the model in order to clarify the earlier finding of lifetime comorbidity being a predictor of adherence [33]. Individuals with a lifetime but no current (at the time of index inpatient treatment) diagnosis of any mental disorder, mood disorder, or anxiety disorder (SCID-I) were defined as remitted.

Adjusted R^2^ is reported for linear regression analysis and Nagelkerkes R^2^ for logistic regression analysis.

## Results

### Sample characteristics at baseline

At baseline T1, the 92 RP participants’ mean age was 24.0 ± 6.5 SD, their BMI had reached 17.9 ± 1.3. The 118 control participants averaged 24.2 ± 5.7 years, and their BMI had reached 17.8 ± 1.2 at the end of inpatient treatment T1. This treatment had lasted, on the average, 89.5 ± 32.7 (RP) and 90.4 ± 36.4 days (controls). Percentage of AN binge eating/purging type was 43.5% in RP and 46.6% in the control group. At T1 (baseline), groups did not differ significantly on these or most other variables [33].

### Course and outcome of body weight

In the RP group, 4 of 92 participants (4.3%) became pregnant between T1 (baseline) and T3 (follow-up), three of them during the course of the RP. During the follow-up period, one of 120 controls (0.8%) became pregnant (chi^2^ not significant). For the main analysis of the BMI, we excluded all participants who became pregnant during the 18 months of the study, because pregnancies obviously affect body weight and BMI.

In the completer sample about which we report here, BMI in the RP participants (n = 88) was 17.9 ± 1.3 at T1 (baseline), 18.3 ± 2.8 at T2 (end of intervention) and 18.8 ± 2.6 at T3 (follow-up). In the control group (n = 117), BMI was 17.8 ± 1.2 at T1 (baseline), 17.7 ± 2.1 at T2 (end of intervention) and 18.4 ± 2.6 at T3 (follow-up). There was a statistical trend for group differences at T2 (end of intervention; t = 1.7; p < .10). RP participants increased their BMI from randomization (T1) to the end of intervention (T2) by 0.47 ± 1.51 BMI points, while controls showed a small weight loss of −0.02 ± 2.05 BMI points. The mean BMI difference from randomization (T1) to the end of the follow-up 18 months later (T3) was 0.86 ± 2.3 BMI points for the RP participants and 0.61 ± 2.6 BMI points in controls (t-tests not significant).

**Figure 2:**
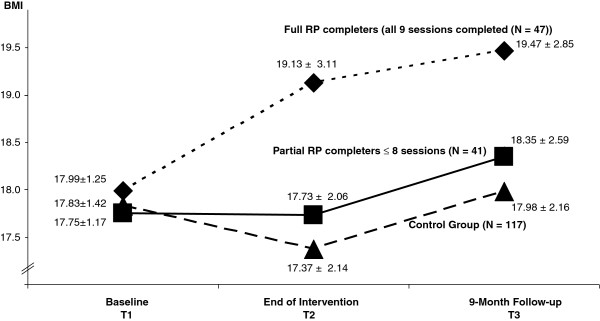
**Course of body weight (BMI) in full RP completers, partial RP completers, and controls, excluding participants who were pregnant at the time of assessment.** Means and standard deviations are indicated for each data point.

A subgroup of RP participants, namely those who took part in every single session of the nine modules of the RP program (full completers), showed a further increase in BMI during the nine-month follow-up period (Figure 2). RP participants who used fewer than all nine modules of the program showed a course of BMI similar to the controls and significantly different from the better course of the participants who adhered strictly to the intervention protocol. In ANOVA, main effects of group (full versus partial completers versus controls) and time as well as the interaction of group by time were significant (F (group) = 5.92, p = .003; F (time) = 10.58, p = .000; F (group by time) = 3.95, p = .004). Pairwise comparison of groups showed full completers reached a significantly (p < .05) higher weight compared to the partial users and the control group at T2 (end of intervention) and at T3 (follow-up). Partial completers and the control group did not differ significantly at any time point, with controls showing the lowest weight.

### Additional treatment

Data on additional treatment for the same time intervals are presented in Table 1.

**Table 1:** Additional treatment between T1 (baseline) and T2 (end of intervention), and between T2 (end of intervention) and T3 (follow-up) in full RP completers, partial RP completers, and control group, excluding participants who were pregnant at the time of assessment

	**Full RP completers**	**Partial RP completers**	**Control group**	**Statistics**
	**N = 47 Mean (SD)**	**N = 41 Mean (SD)**	**N = 115 Mean (SD)**	**F (df = 2)**
**Inpatient treatment between T1 and T2 (weeks)**	0.16 (0.75)^a^	0.92 (2.21)^b^	0.25 (1.18)^a^	4.2
				p = .016
**Inpatient treatment between T2 and T3 (weeks)**	0.69 (2.68)^a^	3.31 (1.08)^b^	2.05 (5.15)^ab^	2.86
				p = .060
**Outpatient psychotherapy between T1 and T2 (sessions)**	16.13 (12.83)^a^	17.54 (14.76)^a^	17.08 (11.75)^a^	ns
**Outpatient psychotherapy between T2 and T3 (sessions)**	15.32 (18.00)^a^	19.39 (23.32)^a^	17.10 (16.80)^a^	ns

ns = not significant.

^a, b,^ Different letters after the means indicate groups that, according to post hoc-Scheffé-tests, differed significantly (p < 0.05) from one another.

Partial RP completers received the highest amount of inpatient treatment during our observation periods, thus differing from both full RP completers and controls who did not differ from each other. No differences were found for intensity of outpatient treatment.

Use of any psychotropic medication during and after the RP was low and did not differ significantly between groups.

### Course of symptoms over time

In both groups (RP and controls), scores of all subscales of the *Morgan Russell Outcome Assessment Schedule* (*MROAS*) changed significantly over time (Table 2). Main group effects were found for “Nutritional Status” and “Mental State”, indicating a generally better adjustment in RP participants. Although the BMI improved in both groups during the RP and follow-up periods, the expert rating of the patient report on nutritional status deteriorated somewhat at the same time. Separate analyses of the single items of this scale showed this to be primarily due to a deterioration in the item “Dietary Restriction”. The items “worry about body weight or appearance” and “body weight” showed only minor variations over time. For the subscale “Menstrual Function”, the main group effect was not significant; there was, however, a significant interaction of group by time effects. RP participants showed a lower level of menstrual function on this scale at the beginning of the intervention and reached a menstrual function comparable to the controls at follow-up.

**Table 2:** **Morgan Russell Outcome Assessment Schedule** (**MROAS**)

**Mean(SD)**	**Relapse prevention (RP)**			**Control group**			**Statistics**		
	**N = 92**			**N = 116**					
	***T1 (Discharge Baseline) *** **Mean (SD)**	***T2 (End of RP) *** **Mean (SD)**	***T3 9- month follow- up *** **Mean (SD)**	***T1 (Discharge Baseline) *** **Mean (SD)**	***T2 (End of Inter-vention) *** **Mean (SD)**	***T3 9- month follow- up *** **Mean (SD)**	***F (group)***	***F (time)***	***F (group x time)***
**Nutritional Status**	9.61 (1,4)	8.75 (2,8)	8.93 (8.9)	9.43 (1.6)	8.19 (2.7)	7.99 (2.7)	4.8	21.8	ns
							p = .029	p = .000	
**Menstrual function** (**N** = **87**/**114**)^**1**^	4.34 (5.3)	6.51 (5.5)	7.94 (5.4)	6.07 (5.7)	7.48 (5.4)	7.54 (5.3)	ns	19.5	3.3
								p = .000	p = .037
**Mental state** (**N** = **92**/**115**)	8.64 (1.9)	8.35 (2.9)	7.97 (2.0)	7.76 (2.2)	7.57 (3.1)	7.20 (2.0)	12.0	4.4	ns
							p = .001	p = .013	
**Sexual adjustment**	7.82 (2.5)	8.90 (2.4)	9.18 (2.8)	7.69 (2.7)	8.81 (2.5)	8.72 (3.0)	ns	27.9	ns
								p = .000	
**Socioeconomic status**	9.29 (1.8)	9.74 (2.2)	10.09 (1.8)	9.23 (2.0)	9.79 (2.0)	10.00 (2.0)	ns	15.9	ns
								p = .000	

^**1**^ Excluding participants who were pregnant at the time of assessment.

ns = not significant.

The *Eating Disorder Inventory* (*EDI*) subscale “Maturity Fears” (F = 4.63; p = .010) revealed a significant group by time interaction, and a statistical trend for EDI “Social Insecurity” (F = 2.87; p = .058). Over time, “Maturity Fears” in RP participants (N = 83) increased from 4.04 ± 3.44 (T1) to 4.33 ± 3.95 (T2) and 4.33 ± 3.98 (T3). In controls (N = 107), scores for “Maturity Fears” over time were 4.24 ± 3.94 (T1), 5.89 ± 4.88 (T2) and 5.69 ± 4.98 (T3). For the RP participants (N = 83), the EDI-scores for “Social Insecurity” were 4.69 ± 3.86 (T1), 5.73 ± 3.79 (T2) and 5.19 ± 4.29 (T3); for the control group (N = 106) the corresponding scores were 4.49 ± 3.66 (T1), 6.48 ± 4.20 (T2), and 6.24 ± 4.73 (T3). The same pattern of results but with more deterioration of symptoms in controls, was observed in both subscales. In other subscales of the EDI, ANOVA revealed significant (p < .05) main effects of time but no significant effects of group or group by time interactions.

Table 3 presents data for the subscales of the expert interview of the *Structured Inventory for Anorexic and Bulimic Syndromes* (SIAB-EX) over time. Comparing profiles of RP and controls, no specific pattern of change could be identified, but generally, data for RP participants showed slightly more favorable course (lower scores) of recovery.

**Table 3:** Scales of the Structured Inventory for Anorexic and Bulimic Syndromes-Interview (Expert-rating SIAB-EX) over time

	**Relapse prevention (RP)**			**Control group**			**Statistics**		
	**N = 92**			**N = 116**					
	***T1 (Baseline = Randomiza tion) Mean (SD)***	***T2 (End of RP) Mean (SD)***	***T3 (9- month Follow -up) Mean (SD)***	***T1 (Baseline = Randomiza tion) Mean (SD)***	***T2 (End of Intervention) Mean (SD)***	***T3 (9- month Follow- up) Mean (SD)***	***F (group)***	***F (time)***	***F (group x time)***
**Body Image and Slimness Ideal** (**BI**)^**1**^ (**N** = **88**/**105**)	1.04 (0.4)	0.97 (0.5)	1.12 (0.6)	1.15 (0.4)	1.11 (0.5)	1.33 (0.6)	6.5	12.7	ns
							p = .012	p = .000	
**General Psycho**-**pathology and Social Integration** (**GenPsySoc**)	0.61 (0.3)	0.47 (0.4)	0.51 (0.4)	0.69 (0.4)	0.51 (0.4)	0.54 (0.5)	ns	20.8	ns
								p = .000	
**Sexuality** (**Sex**)	2.24 (1.2)	1.68 (1.1)	1.60 (1.2)	2.07 (1.2)	1.80 (0.3)	1.74 (1.4)	ns	18.4	ns
								p = .000	
**Bulimic Symptoms (Bul)**	0.34 (0.3)	0.52 (0.6)	0.44 (0.6)	0.33 (0.3)	0.80 (0.9)	0.59 (0.8)	4.1	21.1	4.2
							p = .045	p = .000	p = .015
**Inappropriate Compensatory Behaviors to Counteract Weight Gain**, **Fasting and Substance Abuse** (**Counteract**)	0.04 (0.1)	0.16 (0.1)	0.23 (0.1)	0.06 (0.1)	0.20 (0.2)	0.28 (0.2)	7.6	146.9	ns
							p = .006	p = .000	
**Atypical Binges (AtypBinge)**	0.04 (0.2)	0.17 (0.3)	0.14 (0.3)	0.08 (0.3)	0.22 (0.4)	0.16 (0.3)	ns	12.9	ns
								p = .000	

^**1**^ Excluding participants who were pregnant at the time of assessment.

Ns = not significant.

In ANOVA, the group by time interaction was significant for bulimic symptoms. Breaking down the course of bulimic symptoms into full and partial completers yielded mean values of SIAB-EX bulimic symptoms for full completers (N = 48) of 0.31 ± 0.30 (T1), 0.51 ± 0.65 (T2) and 0.33 ± 0.38 (T3). In partial completers (N = 44), the means were 0.37 ± 0.40 (T1), 0.52 ± 0.67 (T2) and 0.56 ± 0.71 (T3). The ANOVA including full and partial completers and controls (means for controls are presented in Table 3) resulted in F(group) = 2.47, p = .087, F(time) = 12.83, p = .000, and F(group by time) = 2.70, p = .030. A t-test showed significant results (p < .05) for full completers versus controls at T2 and T3, and for partial completers versus controls at T2. Differences for full versus partial completers did not reach significance in t-tests.

### Prediction of weight change in RP participants

Entering the same predictors identified in the ITT sample (for a list of predictors see Table 4) in an analysis of the extended primary outcome covering change in BMI from T1 (randomization) to T3 (follow-up) the SIAB-EX subscale compensatory behavior, additional inpatient therapy, and age at onset of eating disorder had no longer-term predictive value for primary outcome, while adherence to the study protocol, the BIS-11 subscale motor impulsiveness, and the EDI subscale ineffectiveness still exerted a significant impact on primary outcome at T3 (Table 4).

**Table 4:** **Results of multiple linear regression on primary outcome (weight change (BMI) from T1 (baseline) to T3 (follow-up)) in the RP group (replication of ITT analysis **[33]** for the completer sample^1^) Adjusted R^2^ = 0.15; N = 88**

**Predictor**	**Regression coefficient**	**Stand. beta**	**p**
SIAB-EX subscale compensatory behavior T1	- 5.23	- 0.14	.194
BIS-11 motor impulsiveness T1	1.65	0.31	.006
Adherence to RP	1.32	0.28	.010
EDI Ineffectiveness T1	0.15	0.31	.006
Additional inpatient therapy during RP (weeks)	- 0.03	- 0.02	.865
Age at onset of eating disorder	- 0.04	- 0.10	.366

^1^ Excluding participants who were pregnant at the time of assessment.

SIAB-EX = Structured Inventory for Anorexic and Bulimic Eating Disorders, Expert Version.

Subscale compensatory behavior is an abbreviation for ‘inappropriate compensatory behaviors to counteract weight gain, fasting and substance abuse’.

BIS-11 = Barratt Impulsiveness Scale.

EDI = Eating Disorder Inventory 2.

### Prediction of adherence to the RP protocol

Bivariate comparison of T1 (randomization) variables between individuals with good and poor adherence identified only a small number of significant differences. Patients showing good adherence had experienced a later onset (18.4 ± 7.2 versus 15.2 ± 4.0 years, t = 2.6, p = .011) and a shorter duration (5.9 ± 4.4 versus 8.6 ± 4.8 years, t = 2.8, p = .007) of their eating disorder, plus less additional inpatient treatment during the RP (0.2 ± 0.7 versus 0.9 ± 2.1 weeks, t = 2.1, p = .044; good and poor adherence respectively). More of any lifetime mental comorbidity was found in patients with good adherence (79.2% versus 59.1% in poor adherence; chi^2^ = 4.4, p = .037), and patients with good adherence had reported less current anxiety disorders at index inpatient treatment (12.5% versus 31.8% in poor adherence; chi^2^ = 5.0, p = .025). Patients with good and poor adherence did not differ regarding BMI, medication, additional outpatient psychotherapy during the RP, and psychometric measures.

The final logistic regression model identified four predictors of good adherence (Table 5). Remission from mood and anxiety disorder before the index inpatient treatment increased the probability of good adherence. A longer duration of eating disorder and a longer time in additional inpatient treatment during the course of the RP decreased the probability of good adherence.

**Table 5:** Results of logistic regression analysis on good adherence to the RP

**No**	**predictor**	**Wald-statistics**	**Odds ratio**	**95% CI**
1	Remission from lifetime mood disorder at index inpatient treatment	8.4**	5.45	1.73-17.14
2	Remission from lifetime anxiety disorder at index inpatient treatment	4.1*	4.53	1.04-19.73
3	Shorter duration of eating disorder until admission to index inpatient treatment	13.5**	0.79	0.70-0.90
4	Additional inpatient treatment during the course of the RP	4.8*	0.59	0.37-0.94

R^2^ = 0.37 (GOF: Chi^2^ = 4.5; df = 8; p = .81.)

* = p < .05; ** = p < .01.

## Discussion

It still is a difficult task to treat patients with anorexia nervosa successfully. Even intensive in- and outpatient treatments have only limited success in the long run. Therefore, even less must be expected from time-limited, largely automated intervention delivered via DVD or internet. Our patients in the RP group mainly received nine monthly internet sessions supported by (largely automated) e-mails and text messages. Much would already be achieved when body weight at discharge from inpatient treatment would only be maintained. In our study, the RP participants showed an even higher BMI than controls at T2 (end of intervention). During the nine-month follow-up period, both groups (RP and controls) achieved an increase in body weight (BMI), and at the end of the follow-up period, the BMI in the RP group was slightly, but non-significantly higher than in the control group. Thus, when we compare RP completers with controls, the significance of the differences in favor of the RP were not maintained over the full 9-month follow-up period.

However, the results look very different when we break down the group of RP completers into full and partial completers. Full RP completers who participated in all 9 monthly internet sessions showed significant improvement during the intervention period and further significant improvement during the nine-month follow-up. On the other hand, the BMI of the partial RP completers did not differ in its course from the control group. All-or-nothing thinking constitutes a frequent attitude in AN patients. The *full RP completers* seemed to have been fully identified with the aims of the study and were eager not to miss any session. They were the most motivated participants and really wanted to achieve something.

In the long run, partial RP completers and controls were also able to increase body weight but less than the full RP completers. During the same time period, partial RP completers and controls (the latter non-significantly) received more additional inpatient treatment after the end of the intervention period. Inpatient treatment is helpful but the health insurance will only pay when there is a need for it such as in the case of relapse. The higher use of inpatient services in partial completers and controls during the follow-up period can therefore be interpreted as an indicator for relapses. Inpatient treatment in these two groups presumably helped them gain weight. This fact, in turn, made it more difficult for the statistical analyses to uncover the true effects of the online relapse prevention program when comparing the three groups (full completers, partial completers and controls).

Two patterns of predictors of adherence to the RP emerged from regression analysis. One pattern was the chronicity of the eating disorder as defined by duration of eating disorder and additional inpatient therapy during the RP period. Both predictors decreased adherence. The other pattern was the experience of remission from a mental disorder other than eating disorder, which actually increased adherence. A recent review found the evidence on predictors of drop-out from internet-based treatment for psychological disorders to be limited and stated a need for more research in this area [64]. Our findings add to the well-established knowledge on the importance of comorbidity when treating eating disorders; they underline the importance of remission from a comorbid mental disorder, shedding new light on possible factors that influence longer-term outpatient adherence favorably.

Symptom severity did not predict adherence to relapse prevention; consequently, this factor probably assumes a differential role in the assessment of general outcome of AN index treatment in contrast to the period of relapse prevention. As our baseline assessment occurred at the end of intensive inpatient treatment, AN severity was certainly much reduced in our sample (BMI values > 17). The study protocol did not provide for any assessment at the beginning of inpatient treatment, and thus no data were available for this interesting research question.

All values of the SIAB-EX interview subscales at T2 (end of intervention) and at T3 (end of follow-up) were less pathological for the RP as compared to the control group, but except for the subscale “Bulimic Symptoms”, the differences of symptoms between groups did not reach statistical significance. The course of bulimic symptoms was similar to the course of body weight for full completers, showing more improvement than for the control group. The course of bulimic symptoms for partial completers was similar to that of controls; the difference of the scores for full and partial completers did, however, not reach significance.

At T3 (end of 9-month follow-up period), outcome according to the Morgan Russell Outcome Assessment Schedule was more positive in RP participants. However, group by time interactions only reached statistical significance for the subscale “Menstrual Function”.

From the ratings of the clinical interviewers, a pattern of better course and outcome in RP participants emerged. Although statistical significance was rarely reached (for a comment on power and sample size see below), an argument for the positive effect of the RP could be made. The clinical interviewers at T2 (end of intervention) and T3 (follow-up) were blind to the study arm the participant was randomized into, and they did not know data from earlier assessments or patient self-ratings.

The pattern of a better course in RP participants was also found in self-rated maturity fears and social insecurity. However, both scales showed an increase over time in both groups, denoting more fears and insecurity. Chronic AN in itself constitutes a withdrawal from many normal life activities, sometimes lasting for years before initial contact with professionals, and 3 months of inpatient treatment provided a rather protected environment for our study participants. This often combines to years of non-participation in normal life activities. Our post-inpatient assessment period, in contrast, must be considered relatively short in view of the typical history of AN. As normal weight levels are approached, it seems plausible that these young women initially experience *more* fears with regard to entering or reentering a life with adult responsibilities.

Since this is the first internet-based randomized controlled trial for relapse prevention in adult anorexia nervosa, we are only at the very beginning of understanding *predictors for a favorable course* and risk factors for an unfavorable outcome. Only three of six predictors of weight gain during the RP were found to be still relevant for longer-term prediction at follow-up in full and partial completers, after omitting patients who used the RP only marginally or not at all. Short-term predictors related to symptomatic behavior such as compensatory strategies, or additional inpatient treatment and age of onset, were no longer relevant for longer-term prediction. Cognitive characteristics such as “motor impulsiveness”, which in this context means more spontaneity, “ineffectiveness” reflecting poor self-esteem, and adherence to the RP, presumably reflecting among other aspects more motivation to change, constitute short and longer-term predictors indicating the importance of more healthy cognitive attitudes for reaching sustained improvement from anorexia nervosa. The finding of more “ineffectiveness” leading to a better outcome does not necessarily contradict this statement. Possibly, participants with lower self-rated self-esteem were more apt to accept therapeutic guidance, even if this variable was no predictor of adherence.

The study has some *limitations*: 1. Anorexia nervosa is a tough illness to treat, and a cure can hardly be expected. Although the sample of 258 AN patients, randomized into the RP and control conditions, may appear sufficiently large, it apparently was not powerful enough to show many consistent, significant results. In many instances the scores at T3 (follow-up) were less pathological for RP when compared to controls. Only on some subscales were the differences at the follow-up statistically significant in favor of the RP participants. Thus, the study, considering the severity of illness and the difficulties of treatment, was most likely underpowered (in spite of moderate drop-out rates). 2. We possess limited knowledge about a possible selection bias concerning the sample. Patients were consecutively admitted to 8 hospitals in Germany specialized in the treatment of eating disorders. However, the hospitals varied in their recruiting rate for the study. Not all eight hospitals offered a behavioral treatment approach; therefore not all patients were equally prepared for a CBT relapse prevention approach. The drop-out rate among those who received the relapse prevention program (N = 31) was higher than among the controls (N = 6). The most likely reason for this differential drop-out rate is that taking part in the intervention took time and meant investing time and effort. In addition, the RP-participants took part in the assessments at several time points. The latter was true for the controls as well, but the controls did not undergo any intervention program from our group from which they might have dropped out. 4. This study mostly presents results from the completer and not the intention-to-treat (ITT) sample. However, we did also analyze key variables for the ITT sample and found only minor differences as compared to the completer analysis. Course of body weight including data shown in Figure 2, and course of the subscales of the Morgan Russell Outcome Assessment Schedule, EDI and SIAB-EX were very similar in the completer and ITT samples. 5. In our study, we can only evaluate the overall global effects of all treatment components. Analyses of our data do not allow to evaluate the impact of single components such as text messages, e-mail support, the internet program module and the largely standardized telephone contacts with the aim of motivating patients to stay with the program. 6. Having used the latest internet technology, it would have been very desirable to also assess issues concerning cost effectiveness of RP as compared to TAU, similar to the work done by the group of Ruth Striegel-Moore on conventionally offered, guided self-help for binge eating [65,66]. However, time and budget restrictions did not allow for inclusion of this important issue. 7. For a small number of participants we could not obtain a measurement of current weight at T2 (end of intervention) or T3 (follow-up) and had to rely on patients’ self report of their weight. Therefore it can be argued that self-reported body weight was lower than it actually was. However, analyses of data for those patients who both self-reported their body weight and were also weighed by their GP found correlations of .97 and .98 for the BMI. It therefore appears appropriate to include the few cases with self-reported body weight.

The strength and potential of the study are: 1. It is the first internet-based RCT using a CBT approach for relapse prevention in anorexia nervosa. 2. It is one of the few RCTs that targets health improvement in AN patients who previously had required inpatient treatment. 3. It constitutes the only study reporting on the longer-term effects of such an intervention. We not only have data for the time before and after the intervention, but also presented data on the nine-month follow-up. 4. Randomization was quite successful: At the start (baseline), there was no difference between the RP and the control groups for practically all variables assessed. 5. The drop-out rate in our study, when compared to other AN treatment studies, was quite moderate (!). However, there was a considerable number of partial completers in our RP program. A very high drop-out rate has been a problem in quite a few other randomized controlled trials with anorexia nervosa patients. Halmi et al. [38] published important data on the drop-out problem in the McKnight-Study; the authors decided not to publish the data on the participants of the study because the drop-out rate was extremely high. An RCT on online treatment of bulimic symptoms (not anorexia nervosa) reported a drop-out rate from internet treatment of 26% [22], which is comparable to our drop-out rate. 6. Since change in BMI was of importance in our study, we took great pains to measure height and weight professionally rather than relying on self-report of body weight. BMI upon admission and discharge was measured by hospital nurses blind to the design of the study. The randomization at T1 (baseline) occurred when patients were still in the hospital. At T2 (end of intervention) and at T3 (follow-up), patients lived in their home environment, and body weight was usually measured by the general practitioner blind to the design of the study, who then transmitted the data directly to us.

We found a higher rate of pregnancy in the RP group. Conception is based on many factors, and we did not collect data to explore this issue further. The social and biological aspects of pregnancy in or right after remission from AN merit attention, and future studies should be designed to include these aspects.

## Conclusions

In conclusion, our follow-up data indicate that RP as well as controls showed further improvement in body weight (BMI), eating attitudes, and eating behaviors. In the same time period, general psychopathology remained broadly stable on the level achieved at the end of intervention. There was a general tendency for the RP intervention group, as compared to the control group, to show more improvement, but only a limited number of statistical tests actually reached significance. Some areas deteriorated slightly. Considering the chronicity of AN in general, the modest effects of current face-to-face treatments for this disorder and the fact that all our participants presented with a severity of illness that required inpatient treatment, the effects of the relapse prevention program via internet were remarkable. This was especially true for those who made full use of the RP. They reached a significantly higher BMI at follow-up than the controls. Future researchers and clinicians will have the task of predicting which patients they should include in internet-based interventions. Considering our results, it seems rather important to select those who will adhere to the program and will stay with it all the way. For practical purposes based on our data, it would be advisable to offer internet-based relapse prevention programs to patients truly motivated for maintaining the changes achieved through a preceding intensive treatment.

## Endnote

^a)^ In two previous publications [33,55] we used the term “treatment as usual” (TAU) for what we describe as “control group” here. The latter term describes the control condition much better. Patients randomized into the “control group” received no treatment whatsoever within our randomized control trial. Due to ethical considerations, neither the control group nor the RP group could be prevented from seeking additional treatment during the study period but outside the study context. This fact was assessed in detail as an independent variable.

## Abbreviations

BSI: Brief symptom inventory; BMI: Body mass index; BIS-11: Barratt impulsiveness scale; EDI: Eating disorder inventory-2; ITT: Intent-to-treat; MROAS: Morgan Russell outcome assessment schedule; PSR: Psychiatric status rating for Anorexia Nervosa; SCID-I: Structured clinical interview for DSM-IV axis I disorders; SIAB-EX: Structured inventory for Anorexic and bulimic syndromes – expert rating.

## Competing interests

The authors declare that they have no competing interests.

## Authors’ contributions

MMF is principal investigator of the study, funded by the German Department of Research and Technology. He originally formulated the topic of the study, developed the main design (RCT), was the primary investigator of the study and general supervisor. He and NQ made substantive intellectual contributions to the conceptual design, statistical analyses, and interpretation of the data. Both took the lead for drafting the manuscript and contributed essentially to all parts of the paper’s content. SL also has made significant intellectual contributions to the second part of the study and to the data interpretation; she was involved in drafting the manuscript and contributed substantially. All authors and co-authors approved the final version to be published. Each author participated sufficiently in the work to take public responsibility for his or her respective parts of the article.
